# Invited Commentary: Management of Low Colorectal/Coloanal Anastomotic Leak: Results of a French National Inter‐Groups Practice Survey

**DOI:** 10.1002/wjs.12645

**Published:** 2025-05-30

**Authors:** Yasuhiro Ishiyama, Yasumitsu Hirano

**Affiliations:** ^1^ Department of Gastroenterological Surgery Saitama Medical University International Medical Center Hidaka Japan

Anastomotic leakage (AL) persists as the most significant postoperative complication following low anterior resection (LAR) or coloanal anastomosis (CAA) in the surgical management of rectal cancer. Despite considerable advancements in surgical techniques and perioperative management protocols, AL continues to adversely affect both immediate postoperative outcomes and long‐term oncological and functional results. Contemporary literature demonstrates that the incidence of this complication remains at approximately 10%–15%, even within high‐volume tertiary referral centers with specialized colorectal units [1, 2, 3].

Although various diagnostic modalities—such as C‐reactive protein (CRP) monitoring [4], high‐resolution computed tomography (CT) [5], early‐onset pain as a clinical predictor [6], and drain‐fluid amylase measurement [7, 8]—have proven useful, clinical practice still lacks standardized guidelines.

Pastier et al. [9] addressed this gap by surveying 203 French colorectal surgeons on 24 standardized clinical scenarios and distilling the first consensus‐driven algorithm that spans both diagnosis and treatment of AL in patients with a diverting ileostomy. Four easily monitored warning signs—CRP > 250 mg/L, fever ≥ 38.5°C, tachycardia > 100 bpm, and diffuse abdominal pain—were endorsed as triggers for urgent contrast‐enhanced CT without rectal contrast, considered reliable only from postoperative day 3 in two‐thirds of respondents; once any sign is present, 87% recommend immediate imaging.

The treatment algorithm is equally explicit: isolated extra‐digestive air or uncollected effusion is managed with antibiotics alone in 61%–78% of surgeons; a peri‐anastomotic abscess prompts examination under anesthesia, permitting transanal drainage when indicated, in 70%; negative‐pressure endoluminal vacuum therapy is viewed as useful by a slim majority (51%), reflecting its growing acceptance as an organ‐preserving adjunct; and drains are removed when follow‐up imaging confirms resolution of the collection in 64%, illustrating a structured “image‐guided” endpoint. Nearly all participants (97%) prescribe at least 7 days of systemic antibiotics alongside any drainage modality, highlighting consensus on infection control.

Future reports detailing clinical outcomes—such as the success rates of transanal drainage and endoluminal vacuum therapy, rates of anastomotic reconstruction, and stoma avoidance—will further enhance the value of this work. Given its grounding in real‐world data and applicability to everyday practice, Pastier et al.'s study may play a key role in standardizing anastomotic leak management.

## Author Contributions

**Yasuhiro Ishiyama:** conceptualization, data curation, investigation, project administration, writing – original draft. **Yasumitsu Hirano:** supervision, writing – review and editing.

## Conflicts of Interest

The authors declare no conflicts of interest.
